# Management for iatrogenic femoral pseudoaneurysms by embolisation of the aneurysmal neck from the contralateral femoral artery: A report of five cases

**DOI:** 10.1259/bjrcr.20230016

**Published:** 2023-10-18

**Authors:** Kenichi Kato, Tomohiro Suzuki, Makoto Hamano, Eisuke Mukaida, Kazuya Kawashima, Kunihiro Yoshioka

**Affiliations:** 1 Department of Radiology, Iwate Medical University, Iwate, Japan

## Abstract

Femoral pseudoaneurysms are typically associated with femoral arterial catheterisation. Treating iatrogenic morbidity requires curing the disease, without causing any additional complications. Occlusion of the aneurysmal neck is ideal to seal post-catheterised pseudoaneurysms along with maintaining the femoral arterial flow. However, few reports have suggested neck embolisation for post-catheterised pseudoaneurysms. We describe five cases of iatrogenic femoral pseudoaneurysms in patients successfully treated with embolisation through the aneurysmal neck. This technique may be an alternative therapeutic option in managing femoral pseudoaneurysms.

## Introduction

Iatrogenic femoral pseudoaneurysms generally occur after access to the femoral artery during diagnostic or interventional procedures. In this iatrogenic setting, prompt clinical efforts should be made to avoid severe sequelae. Ultrasound-guided treatment or surgical repair are conventionally applied to manage catheterisation-associated femoral pseudoaneurysms. On the other hand, recent advances in coil embolisation have enabled precise occlusion in small target lesions. This study reports five cases of iatrogenic femoral pseudoaneurysms, managed by embolisation of the aneurysmal neck with the contralateral approach using detachable microcoils.

## Case presentation

A summary of the cases in this cohort is presented in Table 1.

**Table 1.** Patient characteristics

Case	Age	Sex	Diagnosis	Site of pseudoaneurysm	Prior procedure
1	50 s	F	SMA embolism	Right CFA	Prolonged taping compression
2	20 s	F	Postpartum hemorrhage	Right CFA	-
3	80 s	M	AMI	Left CFA	-
4	80 s	F	Myositis	Right SFA	-
5	60 s	M	Cholangiocarcinoma	Right CFA	US-guided compression, para-aneurysmal saline injections

SMA, superior mesenteric artery; AMI, acute myocardial infarction; CFA, common femoral artery; SFA, superficial femoral artery

### Case 1

**Figure 1.**
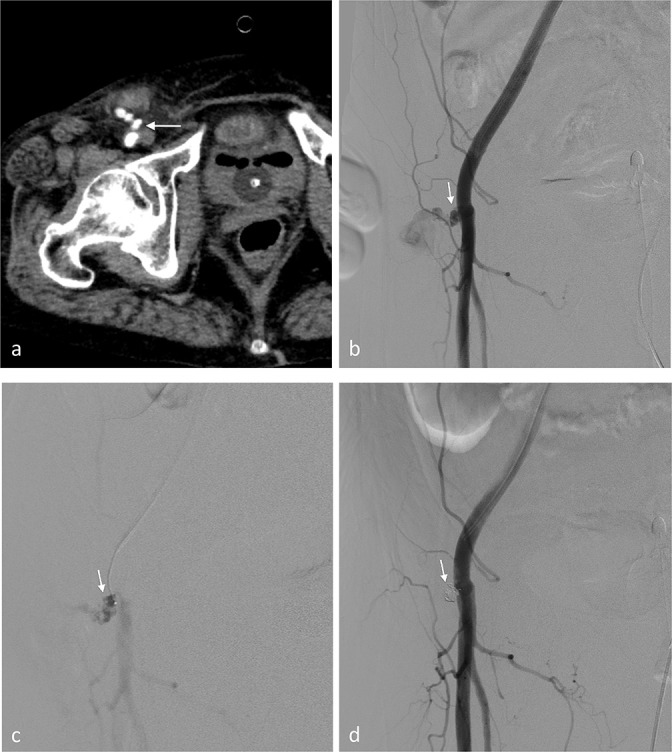
(Case 1) Common femoral pseudoaneurysm after mechanical thrombectomy for a superior mesenteric embolism. (**a**) CE-CT reveals a progressive pseudoaneurysm (arrow) of the right femoral artery. (**b**) Right femoral arteriogram via the contralateral femoral approach. The left anterior oblique (LAO) 50 projection shows right common femoral pseudoaneurysm (arrow). (**c**) The pseudoaneurysm (arrow) is cannulated using a 0.019-inch microcatheter with an angle-shaped tip (Progreatλ; Terumo Corporation, Tokyo, Japan). (**d**) After the deployment of detachable coils (2–2.5 mm diameter; Target, Stryker Neurovascular, CA, USA) into the aneurysmal neck, the final angiogram shows the exclusion of the pseudoaneurysm (arrow).

A female in her 50s was diagnosed with superior mesenteric arterial embolism. Superior mesenteric angiography revealed arterial occlusion, and mechanical thrombectomy was performed using a 6-French aspiration catheter (ThrombusterII, Kaneka Medix Corp, Tokyo, Japan). Reperfusion of the superior mesenteric artery was achieved, and abdominal pain had disappeared. However, femoral pseudoaneurysm was observed on postoperative day (POD) three by contrast-enhanced computed tomography (CE-CT). Hence, right femoral compression was added with bandages. Nevertheless, the pseudoaneurysm increased in size on POD 8. Thus, embolisation was done to achieve haemostasis. The pseudoaneurysm was cannulated from the contralateral femoral artery using a microcatheter (Progreatλ, Terumo Corporation, Tokyo, Japan), and detachable coils were deployed into the aneurysmal neck. Final angiography confirmed the exclusion of the pseudoaneurysm (Figure 1).

### Case 2

**Figure 2.**
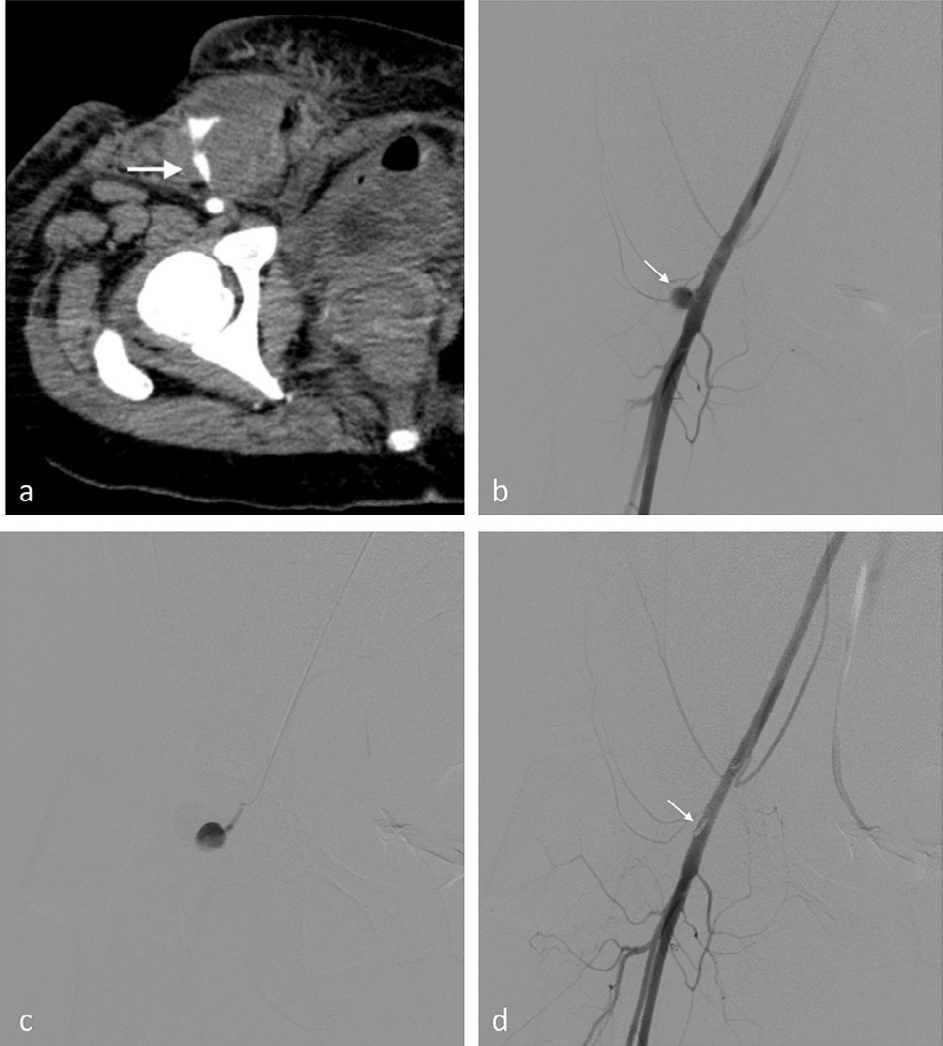
(Case 2) Common femoral pseudoaneurysm after uterine arterial embolisation. (**a**) CE-CT shows a large haematoma with pseudoaneurysm (arrow) despite the manual inguinal compression. (**b**) A pseudoaneurysm (arrow) of the common femoral artery is noted on the right femoral arteriogram. (**c**) The aneurysmal neck is cannulated using a pre-shaped pigtail catheter with cutting of the tip. (**d**) After embolisation of the aneurysmal neck (arrow), the pseudoaneurysm is successfully treated.

An obese female in her 20s underwent uterine arterial embolisation for postpartum haemorrhage associated with uterine atony. The procedure was successful, and the remaining sheath was removed on POD 1. However, a huge uncontrolled haematoma occurred immediately after manual inguinal compression. Endovascular intervention was performed. A pre-shaped pigtail catheter, with a cutting tip (Hanako Medical, Saitama, Japan), was cannulated into the aneurysmal neck via the contralateral femoral artery. After the aneurysmal neck embolisation with microcoils, the pseudoaneurysm disappeared on angiography (Figure 2).

### Case 3

**Figure 3.**
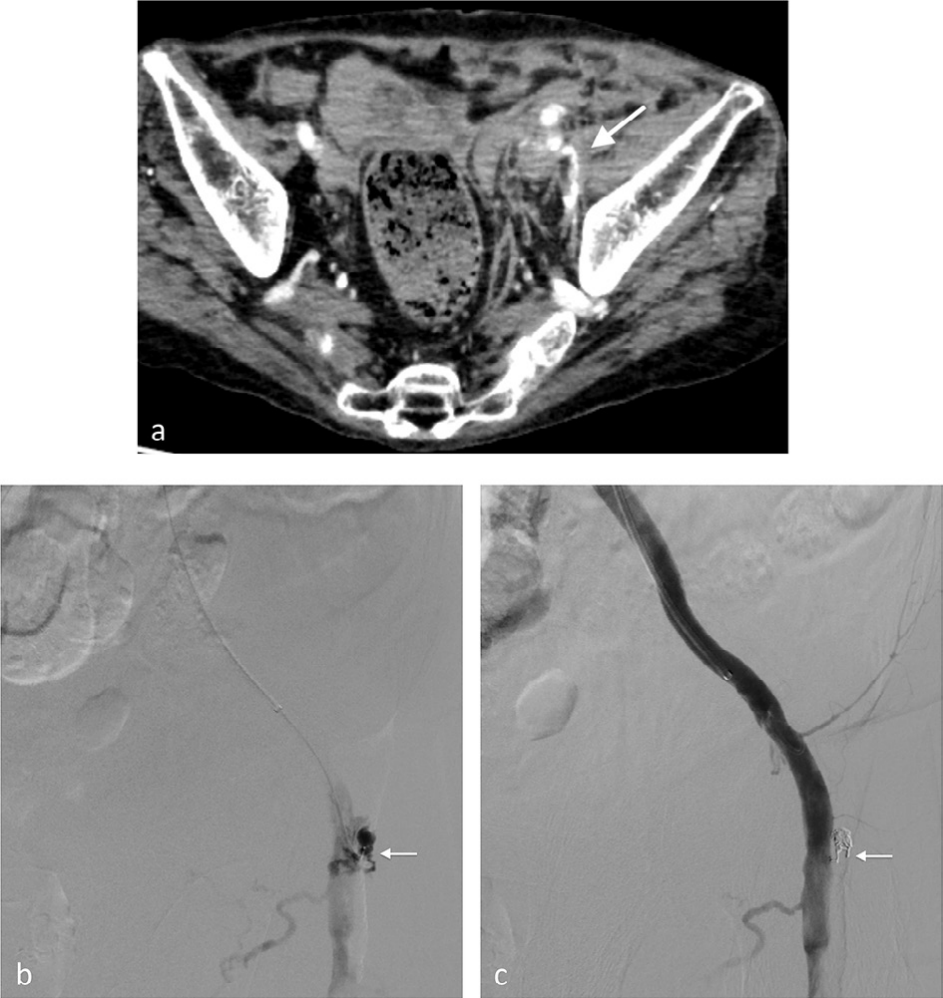
(Case 3) Common femoral pseudoaneurysm after removal of a 14-French introducer sheath. (**a**) Extravasation is noted around the left iliopsoas muscle (arrow). (**b**) Left iliac arteriogram on right anterior oblique (RAO) 40. The pseudoaneurysmal neck (arrow) is cannulated using a microcatheter inserted via right femoral artery. (**c**)The pseudoaneurysm (arrow) is successfully embolised.

A male in his 80s underwent percutaneous coronary intervention with mechanical circulatory support (Impella; Abiomed, Danvers, MA, USA) due to acute myocardial infarction. Although a 14-French introducer sheath was removed using a suture device, retroperitoneal haematoma caused hypotension. Emergent CE-CT revealed a pseudoaneurysm at the puncture site, which extravasated around the iliopsoas muscle. Accordingly, emergent embolisation was planned. A pre-shaped pigtail catheter with a cutting tip (Hanako Medical, Saitama, Japan) and a microcatheter (Progreatλ, Terumo Corporation, Tokyo, Japan) were cannulated into the pseudoaneurysm via the contralateral femoral artery. After embolisation using microcoils, extravasation disappeared (Figure 3).

### Case 4

**Figure 4.**
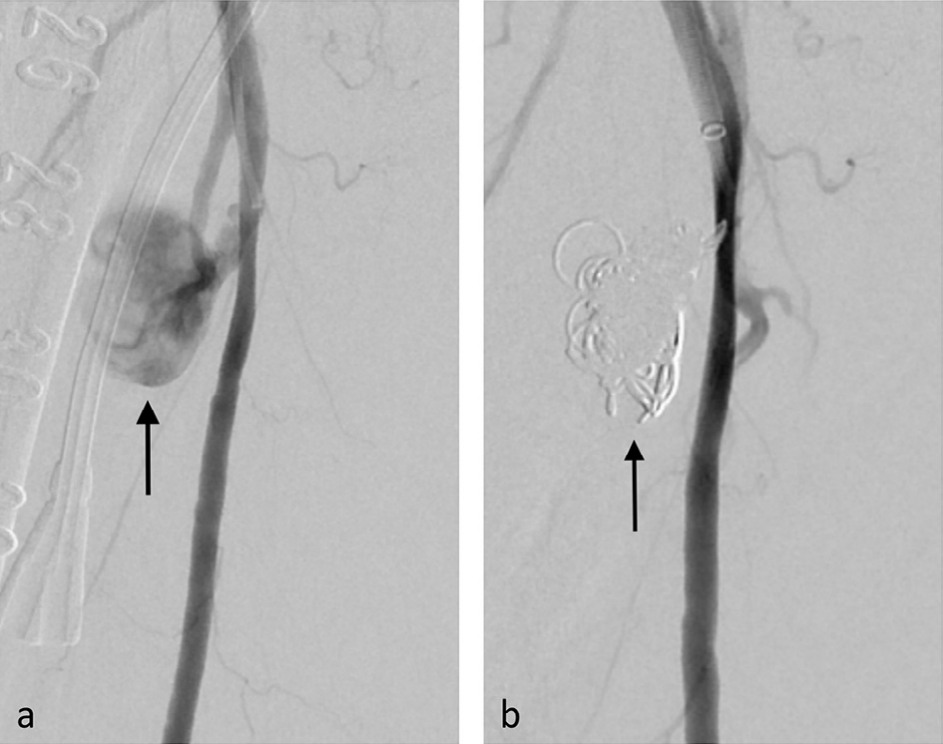
(Case 4) Superficial femoral pseudoaneurysm associated with the catheterisation of the femoral vein. (**a**) Right arteriogram via the contralateral femoral approach. A huge pseudoaneurysm (arrow) from the superficial artery was observed on LAO 24 with slight arterial compression. (**b**) The pseudoaneurysm was cannulated, and aneurysmal sac as well as aneurysmal neck was embolised (arrow).

A female in her 80s was hospitalised due to myositis. Immunoadsorption plasmapheresis was planned, and a double-lumen catheter was introduced via the right femoral vein. One day after the procedure, her anaemia and right thigh swelling progressed. A pseudoaneurysm of the superficial femoral artery was noted on CE-CT. Consequently, embolisation of the pseudoaneurysm was performed via the contralateral femoral artery. The aneurysmal sac as well as aneurysmal neck was embolised, and haemostasis was achieved (Figure 4).

### Case 5

**Figure 5.**
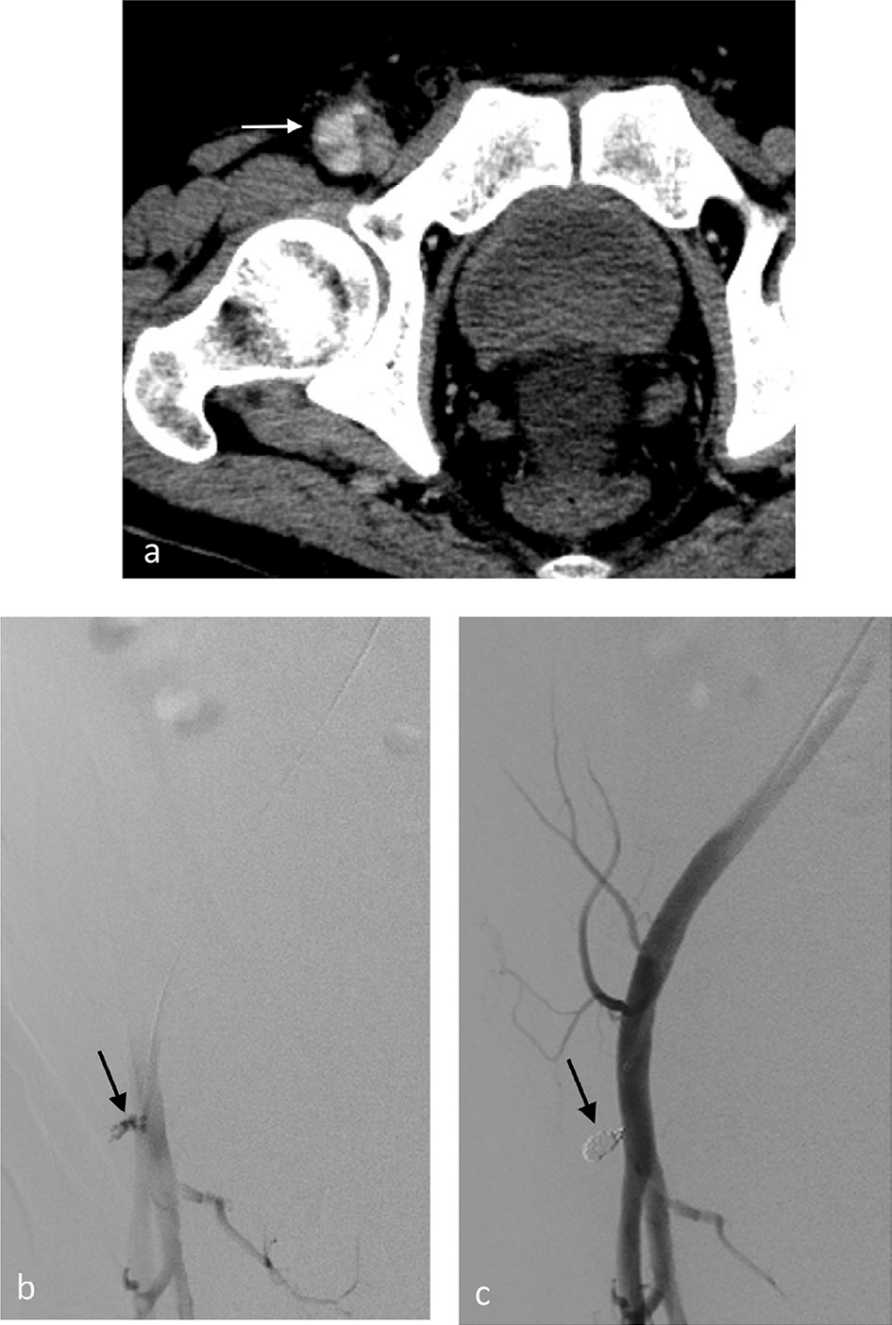
(Case 5) Common femoral pseudoaneurysm after the interventional procedure for postoperative haemorrhage of hepatobiliary surgery. (**a**) Common femoral pseudoaneurysm (arrow) persists over nine days under severe coagulopathy. (**b**) Right femoral arteriogram via the left femoral approach. Pseudoaneurysm (arrow) remains despite US-guided compression and several para-aneurysmal saline injections. (**c**) The pseudoaneurysmal neck was selectively cannulated with a microcatheter (Estream IGT, Toray, Tokyo, Japan), and exclusion of the pseudoaneurysm (arrow) is achieved after the embolisation.

A male in his 60s, with hepatic hilar cholangiocarcinoma, developed postoperative haemorrhage, and underwent embolisation of the right hepatic artery via the right femoral artery. Severe coagulopathy is associated with obstructive jaundice. On POD 9, a follow-up CE-CT revealed a common right femoral pseudoaneurysm. Ultrasound (US)-guided compression and para-aneurysmal saline injections were performed. Although the pseudoaneurysm had partially shrunk, it was residual. Therefore, embolisation of the pseudoaneurysm was performed. The pseudoaneurysmal neck was cannulated using a microcatheter (Estream IGT, Toray, Tokyo, Japan) using left femoral approach. After pseudoaneurysmal embolisation, haemostasis was achieved (Figure 5).

## Discussion

Appropriate catheterisation and careful compression of the puncture site are fundamental to avoid iatrogenic pseudoaneurysms. However, iatrogenic pseudoaneurysms may occur due to various factors, such as anticoagulant therapy, a larger access sheath, and obesity.^1^ Various approaches, including US-guided compression, US-guided thrombin injection (UGTI),^2^ para-aneurysmal saline injections,^3^ and surgical repair, have been utilised to manage pseudoaneurysms. Surgical repair is a reliable management method for achieving complete haemostasis. Nevertheless, the indications for surgical repair are limited and unsuitable for patients with morbidities. Recently, UGTI has been introduced as a non-invasive technique. The success rate of the UGTI is high and can be applied repeatedly. However, complications, such as parent-arterial occlusion or distal embolisation, may occur. Puncturing into the sac may be difficult in cases where there is dominant extravasation with a small pseudoaneurysmal sac. Stent grafting might be an alternative approach to exclude common femoral pseudoaneurysms. However, stent grafting for common femoral pseudoaneurysms is indicated only in selected cases where the graft can be safely deployed without covering the femoral arterial bifurcation. The coil embolisation method involves two possible accesses to the pseudoaneurysm.^4–6^ One method is coil insertion with direct percutaneous puncture into the aneurysmal cavity. Another method is coil introduction into the aneurysmal cavity through a manipulated catheter, usually via the contralateral femoral artery. Our cases highlight a modified strategy to occlude the pseudoaneurysmal flow mainly by embolisation of the aneurysmal neck. The cannulation into the post-catheterisation aneurysmal neck be technically challenging.^6^ Typically, the post-catheterised aneurysmal neck exists in the anteroposterior direction. Optimum oblique projection is essential for the cannulation of the aneurysmal neck and precise deployment of microcoils. The combination of a guiding sheath and angle-shaped guiding catheter, including a pigtail catheter with a cutting tip, is also useful. Moreover, a pre-shaped microcatheter can provide more successful cannulation. Sealing the post-catheterised aneurysmal neck is the key to achieving complete haemostasis to overcome these iatrogenic conditions. However, this study has some limitations. Due to a small cohort, the validity of aneurysmal neck embolisation for femoral pseudoaneurysm could not be evaluated. Complications associated with superficial located coils are also unclear due to the lack of long-term data. Although a larger scale of clinical study needs to be conducted, this technique could be one of the options for treating iatrogenic femoral pseudoaneurysms.

## Learning points

- Femoral iatrogenic pseudoaneurysms require prompt and adequate management.
- Sealing the post-catheterised aneurysmal neck is logical for treating iatrogenic femoral pseudoaneurysms.
- Embolisation of pseudoaneurysmal neck is an alternative therapeutic option for femoral pseudoaneurysms.
